# Dexamethasone with aggressive warming facilitates pain reduction, reduced blood loss, and quicker recovery after total hip arthroplasty

**DOI:** 10.1038/s41598-023-47050-7

**Published:** 2023-11-09

**Authors:** Fulin Li, Xiao Huang, Wenhui Liu, Wenwen Huang, Jinwen Cheng, Dong Yin

**Affiliations:** Department of Joint and Sports Medicine Surgery, The People’s Hospital of Guangxi Zhuang Autonomous Region, Nanning, 530021 China

**Keywords:** Clinical trial design, Medical research

## Abstract

This study aimed to evaluate the optimal frequency of dexamethasone (DEX) administration and the efficacy of DEX with aggressive warming in total hip arthroplasty (THA), which remains unclear. A total of 150 patients were treated with DEX (10 mg) once before and once or twice after surgery with or without intraoperative aggressive warming. On postoperative day 3, the dynamic visual analogue scale scores and C-reactive protein (CRP) and interleukin-6 (IL-6) levels in participants administered with DEX twice after surgery were significantly lower than those who did not receive the second dose. The range of motion (ROM), postoperative fatigue based on Identity-Consequence-Fatigue Scale, average temperature at different stages, intraoperative blood loss, and postoperative drainage volume in patients who were subjected to warming were significantly higher than those who were not. The degree of satisfaction was also higher in the patients who received both second dose and warming than those who received neither. No differences in complications were observed based on the treatments. An additional dose of DEX at 48 h post-surgery has short-term advantages in terms of analgesia, anti-inflammatory effects, and accelerated rehabilitation after THA. DEX combined with aggressive warming further optimises short-term ROM and fatigue and improves the degree of satisfaction.

Clinical trial was registered in the International Clinical Trial Registry, and the date of registration is 2/12/2020 (ChiCTR2000040560).

## Introduction

Total hip arthroplasty (THA) is an effective method for treating advanced hip diseases, such as osteoarthritis and osteonecrosis of the femoral head^1^. However, moderate-to-severe postoperative pain often occurs after THA, and postoperative nausea and vomiting (PONV) often slows postoperative rehabilitation and reduces patient satisfaction^2,3^. Recently, the use of multimode analgesia technology has aided in reducing postoperative pain. However, control of severe pain after THA remains challenging^4^. Inflammation is considered a key factor of postoperative pain^3^. Lately, dexamethasone (DEX) has been shown to effectively control postoperative inflammation, reduce postoperative pain, and reduce the incidence of PONV^5–7^. However, owing to clinical heterogeneity, there is no uniform clinical standard for the optimal frequency of DEX administration in THA. Considering that the half-life of DEX is approximately 24–36 h, some studies indicate that the maximum effect of a single dose of DEX occurs within 24 h^8^. Nevertheless, patients who receive DEX once or twice may continue to experience postoperative pain^9^.

Hypothermia is a common complication that is often overlooked during the perioperative period. Studies have reported that the incidence of hypothermia is as high as 60%. The low temperature of the operating room and various types of anaesthesia and surgical procedures can easily result in hypothermia. Even mild levels of hypothermia can cause serious problems, such as an increased risk of postoperative incision infection, prolonged recovery time after anaesthesia, increased blood loss, deceleration of postoperative rehabilitation, and reduced satisfaction^10,11^.

Sufficient attention must be paid to the issue of hypothermia. The use of aggressive warming combined with DEX in the perioperative period after THA has not been reported. This study aimed to evaluate the efficacy of an additional dose of DEX 48 h after THA and that of aggressive warming combined with DEX in accelerating rehabilitation and improving patient satisfaction.

## Methods

### Patients and design

This study was conducted at the People's Hospital of Guangxi Zhuang Autonomous Region from December 2020 to September 2022 and was approved by the hospital’s Institutional Review Board, including any relevant details and all experiments were performed in accordance with relevant guidelines and regulations. This study was designed as a prospective double-blind, randomised controlled trial that is registered with the Chinese Clinical Trial Registry (ChiCTR2000040560). All patients provided written informed consent before participation.

The inclusion criteria considered unilateral THA and informed consent. Exclusion criteria included allergy to DEX, age ≤ 18 years or ≥ 75 years, use of any glucocorticoids in the past three months or that of any strong opioids within a week before surgery, history of severe heart disease (NYHA > 2), liver or kidney failure, systemic rheumatic diseases (rheumatoid arthritis, ankylosing spondylitis, or systemic lupus erythematosus), hip surgery history, lack of cognitive function and normal sensation, loss of follow-up, opioid dependence, and oral anticoagulation or medication affecting thrombocyte aggregation.

All eligible patients were randomly assigned to groups A, B, or C. All patients were randomly assigned sequences hidden in opaque sealed envelopes that were opened only before surgery. DEX (2 mL, 10 mg) was injected intravenously in group A patients before anaesthesia induction and 24 h after surgery. Normal saline (2 mL) was administered at 48 h. Patients in group B were administered DEX (10 mg) intravenously before anaesthesia induction and after surgery at 24 and 48 h. In group C, the DEX regimen as that for group B was followed with the additional application of aggressive warming during the surgical procedure. The patients, surgeons, anaesthesiologists, nurses, and research assistants who collected the data were blinded to the grouping during the experiment.

### Surgery procedure

All surgeries were performed by a senior professional physician from the surgical team in a hundred-level laminar flow operating room. All patients were evaluated by anaesthesiologists and were administered general anaesthesia in the lateral decubitus position using the anterolateral incision approach and cementless prosthesis. To control for variables, nerve blocks and intravenous analgesia were not administered.

During the aggressive warming, axillary temperature measurements were used to observe changes in the patients' body temperature. Third-party developers assisted in recording temperature changes in the three groups of patients before surgery and at various time points after anaesthesia. Aggressive warming measures included controllable electric heating blankets, fluid warmers, and control of the operating room temperature (24 °C). During the surgery, the temperatures of the controllable thermal blanket and infusion were adjusted according to the changes in the patients' body core temperature, and the temperature was maintained above 36 °C.

### Postoperative care protocol

Ankle dorsal, plantar flexion, and quadricep strength exercises were started in the recovery bay; the patients were subcutaneously injected with low-molecular-weight heparin (LMWH) 8 h after the operation. The patients received a standard supervised daily physiotherapy regimen which included strength training, walking, and intermittent pneumatic compression. All the patients received the same analgesia and PONV management regimen. After returning to the ward, pain was assessed using a VAS ranging from 0 (no pain) to 10 (worst pain imaginable). If the VAS scores were between 4 and 6, oxycodone was administered orally at Q8h (10 mg). If the pain exceeded a level of 6, 100 mg of tramadol was administered intramuscularly. The degree of nausea was assessed by another VAS ranging from 0 (no nausea) to 10 (the worst nausea imaginable). If the nausea VAS score exceeded 5, 10 mg of metoclopramide was administered as the first-line antiemetic rescue; a further dose of 5 mg ondansetron was administered as the second-line antiemetic rescue if nausea persisted after 30 min. The Identity-Consequence-Fatigue Scale (ICFS)^12^ was used to assess fatigue before surgery and on POD3. Deep vein thrombosis was detected using Doppler ultrasound before discharge and one month later.

### Patient blood loss

Intraoperative blood loss (IBL) was based on the volume of saline used to flush the incision during operation, the volume of fluid aspirated by the suction device, and the static weight gain of gauze during operation recorded by a third party. Postoperative drainage volume (PDV) was measured by a third party with a syringe.

### Patient satisfaction

We adopted the Quality of Recovery-40 questionnaire (QoR-40) to assess patient satisfaction. The QoR-40 questionnaire is a universally or externally validated scale for the assessment of patient satisfaction after major surgery based on five recovery criteria: physical comfort, emotional state, physical independence, psychological support, and pain. The QoR-40 questionnaire is graded from 40 to 200, where 40 indicates Terrible, 41 to 119 indicates Not Bad, 120 to 159 indicates Good, and ≥ 160 indicates Wonderful patient satisfaction.

### Postoperative complications

In our study, closed attention was paid to complications during hospitalization and at outpatient follow-up at 1, 2, and 3 months after discharge, mainly including surgical site infections (SSI) or gastrointestinal bleeding (GIB). SSI was mainly based on clinical manifestations, laboratory tests and imaging findings, and histological examination around the prosthesis and intraoperative manifestations were performed when necessary. Acute joint pain, wound inflammation (fever, redness and swelling), joint effusion and loss of function are common local manifestations in acute stage. The systemic manifestations included fever, malaise and nausea. Laboratory tests included C-reactive protein (CRP), erythrocyte sedimentation rate (ESR), interleukin-6 (IL-6), procalcitonin (PCT), and bacterial culture of wound secretion. Imaging examination mainly includes X-ray, CT, MRI and B ultrasound examination. GIB can be diagnosed by hematemesis, melena and positive fecal occult blood test and gastrointestinal endoscopy should be performed if necessary.

### Statistical analyses

All statistical analyses were performed using SPSS version 24 (IBM Corp., Armonk, NY, USA). Results are presented as mean ± standard deviation (continuous) and number (qualitative variables). One-way ANOVA and Tukey's post-hoc test were used to evaluate parametric data, and the Mann–Whitney U-test was used for nonparametric data. Pearson’s Chi-square test or Fisher’s exact test was used to analyse qualitative comparative data. *P* < 0.05 was considered statistically significant. With a power of 0.90 and a significance level of 0.05, 43 patients per arm were required for the study. Considering the dropout rate, the sample size should be increased from 10 to 15%. In this study, a value of 15% was estimated. Therefore, a sample size of 50 patients in each group was required for this trial; thus, the total sample size employed was 150.

### Ethics approval and consent to participate

This study was approved by the Medical Ethics Committee (the People’s Hospital of Guangxi Zhuang Autonomous Region) and the informed consent was obtained from our responsible Investigational Ethics Review Board.

## Results

### Patient demographics

**Figure 1:**
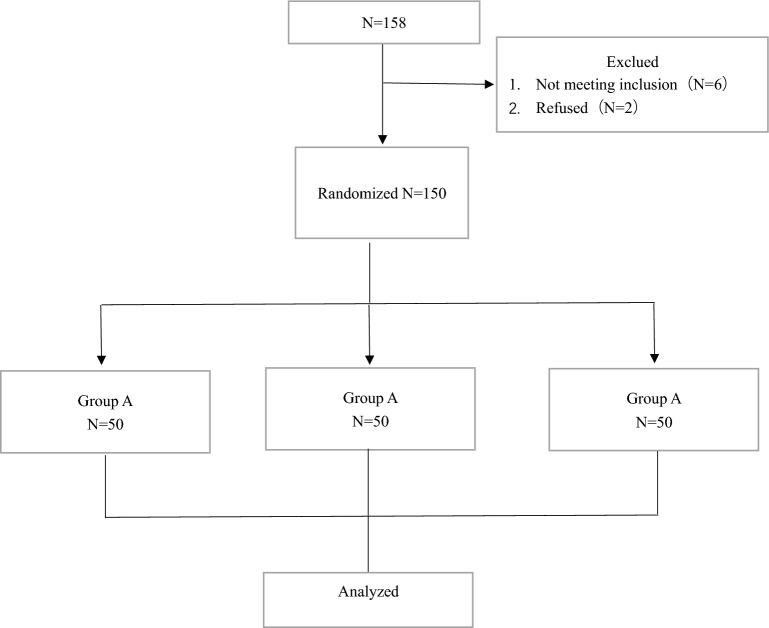
Schematic diagram of the patient study process.

A total of 158 patients were recruited between December 2020 and September 2022. All were scheduled to undergo primary unilateral THA at our medical institution. Among them, six patients did not meet the inclusion criteria, and two refused to participate. The remaining 150 eligible participants were included in the intervention as part of three random groups (groups A–C, 50 participants per group) (Fig. 1). Baseline characteristics and preoperative variables were comparable among the three groups (Table 1).

**Table 1:** Demographic data of the three groups.

	Group A	Group B	Group C	*P* value
N	50	50	50	–
Age (y)	64.02 ± 5.82	64.22 ± 5.46	63.94 ± 4.03	0.96
Gender (M/F)	21/29	14/36	20/30	0.29
Height (m)	1.62 ± 0.08	1.62 ± 0.07	1.63 ± 0.06	0.75
Weight (kg)	64.39 ± 8.30	64.61 ± 6.48	64.46 ± 5.12	0.98
BMI (kg/m^2^)	24.63 ± 2.77	24.73 ± 3.08	24.43 ± 2.30	0.37
Hypertension (Y/N)	11/39	10/40	13/37	0.77
Diabetes (Y/N)	2/48	3/37	2/48	0.86
Etiology (ONFH/OA/DDH)	27/14/9	24/16/10	29/14/7	0.88
Preoperative CRP	7.86 ± 2.67	7.60 ± 2.19	7.92 ± 2.19	0.78
Preoperative IL-6	2.33 ± 1.38	2.48 ± 1.54	2.49 ± 1.34	0.81
Preoperative rest VAS	5.44 ± 0.86	5.34 ± 1.08	5.40 ± 0.90	0.87
Preoperative motive VAS	7.98 ± 0.87	8.04 ± 0.88	7.88 ± 0.80	0.64
Preoperative ICFS score	61.76 ± 5.86	62.58 ± 4.49	63.34 ± 4.46	0.29
Preoperative ROM	90.48 ± 4.10	91.20 ± 3.85	91.24 ± 3.73	0.55
Preoperative Hb	126.12 ± 8.89	126.06 ± 7.04	125.74 ± 6.78	0.97

*BMI* Body Mass Index, *ONFH* osteonecrosis of the femoral head, *OA* osteoarthritis, *DDH* developmental dysplasia of the hip, *CRP* C-reactive protein, *IL-6* interleukin-6, *ICFS* Identity-Consequence-Fatigue Scale, *ROM* range of motion, *Hb* hemoglobin.

### Inflammation markers

**Figure 2:**
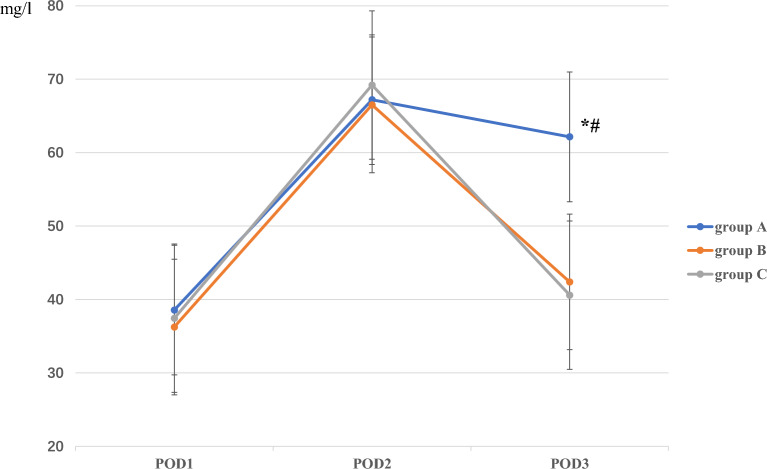
The comparison of CRP among the three groups on POD1, 2, and 3.

The C-reactive protein (CRP) levels in groups B (42.40 ± 7.28 mg/L) and C (40.60 ± 6.48 mg/L) were generally significantly lower than those in group A (62.15 ± 9.51 mg/L; *P* < 0.05) at 72 h postoperatively. This difference was not detected between groups B and C at 72 h postoperatively (*P* = 0.25), and there were no differences among the three groups at 24 or 48 h (*P* = 0.32 or *P* = 0.46, respectively; Fig. 2).

**Figure 3:**
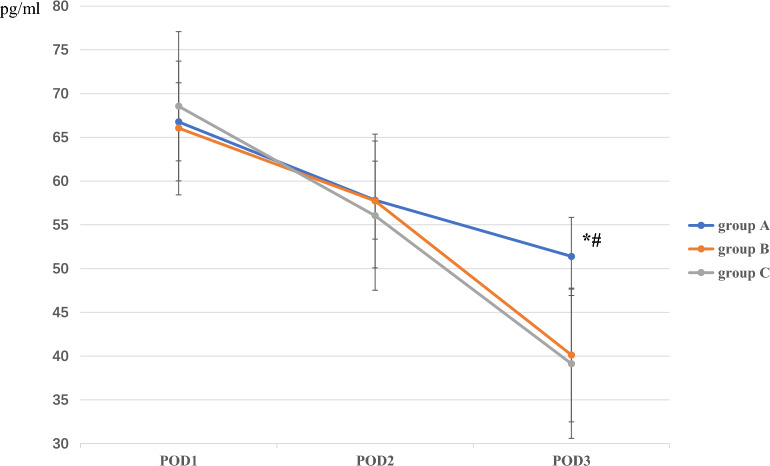
The comparison of IL-6 among the three groups on POD 1, 2, and 3.

Consistent with the differences in CRP level, the interleukin-6 (IL-6) levels in group B (40.13 ± 6.65 pg/mL) and C (39.13 ± 6.32 pg/mL) were generally significantly lower (both *P* < 0.001) than that in group A (51.39 ± 9.13 pg/mL) at 72 h postoperatively. This difference was not detected between groups B and C at 72 h postoperatively (*P* = 0.50), and there were no differences among the three groups at 24 or 48 h (*P* = 0.38 and *P* = 0.49, respectively; Fig. 3).

### Pain level and analgesic rescue

**Figure 4:**
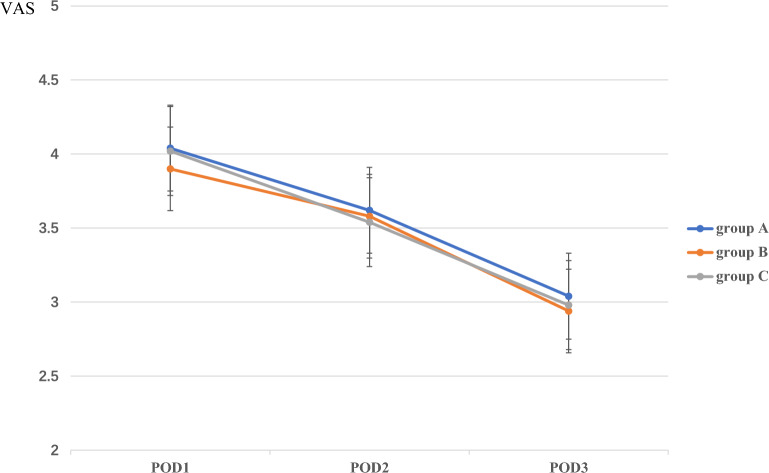
The comparison of VAS of pain at rest among the three groups on POD 1, 2, and 3.

**Figure 5:**
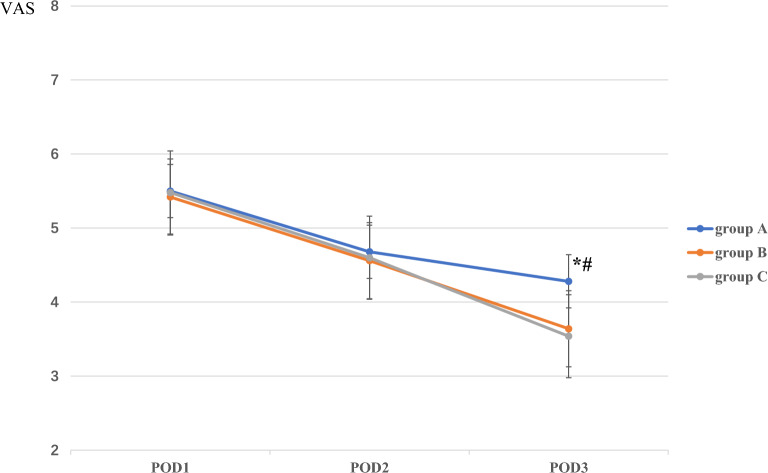
The comparison of VAS of pain at walking among the three groups on POD 1, 2, and 3.

The pain scores at rest and walking on postoperative day (POD) 1 and POD2, and at rest on POD3 did not differ among the three groups. However, the pain scores at walking on POD3 were significantly lower for group B (3.64 ± 0.53, *P* < 0.001) and C (3.54 ± 0.58, *P* < 0.001) than those of group A (4.28 ± 0.70). Such a difference was not detected between groups B and C (*P* = 0.41; Figs. 4, 5).

Detailed data regarding the number of patients requiring oxycodone and tramadol in each group and the cumulative oxycodone and tramadol consumption among all patients in each category are summarised in Table 2. There were no statistically significant differences among the three groups in the number or cumulative consumption of oxycodone or tramadol.

**Table 2:** The requirement of rescue treatment among the three groups.

	Group A	Group B	Group C	*P*	*P*1	*P*2	*P*3
Oxycodone							
N	39/50	35/50	33/50	0.40	0.36	0.18	0.67
Total dose (mg)	740	580	550	0.20	0.16	0.09	0.79
Tramadol							
N	5/50	4/50	4/50	0.92	0.73	0.73	–
Total dose (mg)	800	700	600	0.92	0.84	0.68	0.84
Metoclopramide							
N	4/50	3/50	3/50	0.90	0.70	0.70	–
Total dose (mg)	50	40	30	0.82	0.76	0.53	0.76
Ondansetron							
N	3/50	2/50	2/50	0.86	0.64	0.64	
Total dose (mg)	15	10	10	0.86	0.64	0.64	–

*P*: A versus B versus C; *P*1: A versus B; *P*2: A versus C; *P*3: B versus C.

### PONV and antiemetic rescue

The incidence of PONV was significantly higher in groups A (9 of 50, *P* = 0.025) and B (8 of 50, *P* = 0.045) than in group C (2 of 50). The difference was not statistically significant between groups A and B (*P* = 0.79; Table 3).

**Table 3:** The clinical effect and complications.

	Group A	Group B	Group C	*P*	*P*1	*P*2	*P*3
PONV	5/50	4/50	4/50	0.92	0.73	0.73	–
ICFS	76.46 ± 10.13	71.06 ± 9.19	66.58 ± 8.89	0.00	0.005	0.00	0.02
ROM	93.80 ± 3.88	97.02 ± 2.23	98.82 ± 2.76	0.00	0.00	0.00	0.004
p-LOS	5.10 ± 0.54	4.98 ± 0.43	4.92 ± 0.60	0.23	0.26	0.09	0.57
Wound problems	2/50	3/50	2/50	0.86	0.65	–	0.65
SSI	0/60	0/60	0/60	–	–	–	–
GIB	0/60	0/60	0/60	–	–	–	–

*P*: A versus B versus C; *P*1: A versus B; *P*2: A versus C; *P*3: B versus C.

*PONV* postoperative nausea and vomiting, *ICFS* Identity-Consequence-Fatigue-Scale, *ROM* range of motion, *p-LOS* postoperative length of stay, *SSI* surgical site infection, *GIB* gastrointestinal bleeding.

Detailed data regarding the number of patients requiring metoclopramide and ondansetron in each group and the cumulative metoclopramide and ondansetron consumption among all patients in each category are summarised in Table 2. Fewer patients required metoclopramide in group C (2 of 50) than in groups A (9 of 50, *P* = 0.025) or B (8 of 50, *P* = 0.045), with no statistically significant difference between groups A and B (*P* = 0.79). The overall consumption of tramadol was lower in group C (50 mg) than in groups A (150 mg, *P* = 0.04) and B (110 mg, *P* = 0.23). However, there were no statistically significant differences between groups A and B (*P* = 0.42).

During the study period, there were no differences among the three groups in terms of the number of patients requiring ondansetron (3 in group A, 2 in group B, and 2 in group C; *P* = 0.86) and overall consumption (15 mg in group A, 10 mg in group B, and 10 mg in group C; *P* = 0.86; Table 2).

### Related index of temperature

**Figure 6:**
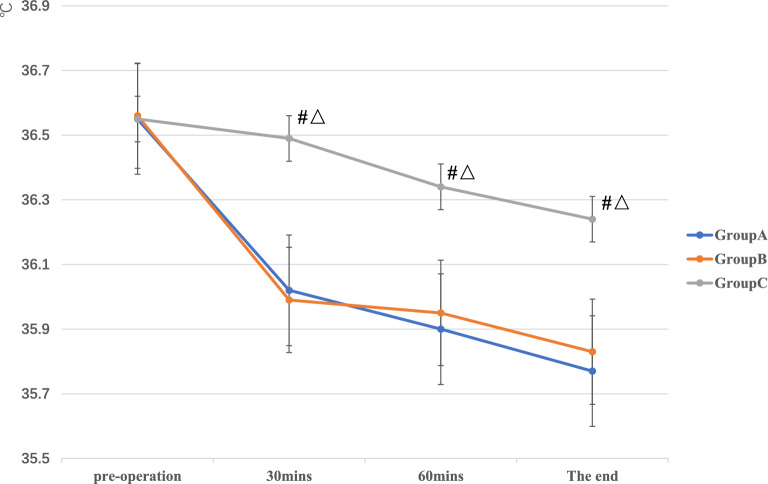
The comparison of temperature at different stages among the three groups.

There was no significant difference in the preoperative temperatures of the participants among the three groups (*P* = 0.98). The temperatures of the participants in the three groups at 30 min, 60 min, and at the end of the operation were statistically significant (*P* < 0.001). Among them, the average temperature of group C patients was 36.49 ± 0.29 °C at 30 min, 36.34 ± 0.28 °C at 60 min, and 36.24 ± 0.28 °C at the end of the operation. These values were significantly higher than those of group A and B patients. There was no significant difference in the temperature between groups A and B at different stages of surgery (*P* > 0.05; Fig. 6).

### Blood loss volume and transfusion rate

**Figure 7:**
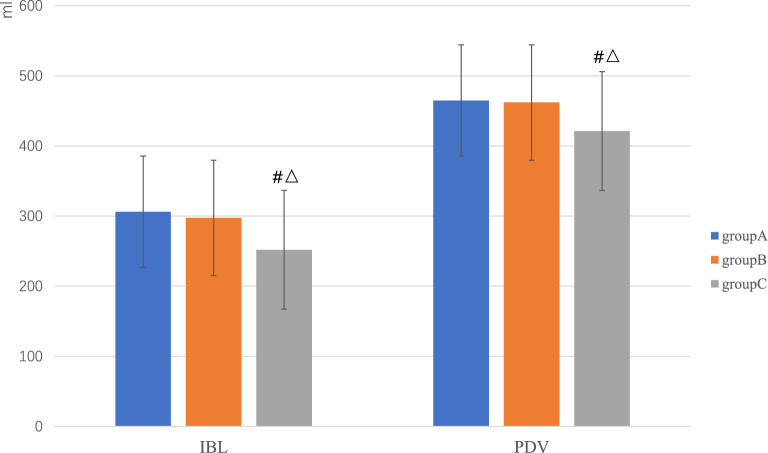
The comparison of blood loss volume among the three groups.

The difference in IBL among the three groups was statistically significant (*P* < 0.001). Among them, IBL was lowest for group C participants (252.00 ± 43.43 mL). This value was significantly lesser than that of group A (306.20 ± 54.39 mL, *P* < 0.001) and group B (282.22 ± 61.97 mL, *P* < 0.001). The difference was not statistically significant between groups A and B (*P* = 0.36; Fig. 7).

The difference in PDV among the three groups was statistically significant (*P* < 0.001). Among them, PDV was lowest in group C (421.30 ± 44.34 mL). This value was significantly less than those of group A (464.96 ± 47.40 mL, *P* < 0.001) or group B (462.00 ± 55.40 mL, *P* < 0.001). The difference between groups A and B was not significant (*P* = 0.76; Fig. 7).

The blood transfusion rates in groups A, B, and C were 6.00% (3/50), 6.00% (3/50), and 2.00% (1/50), respectively, with no significant differences among them (*P* = 0.55).

### Range of motion (ROM), visual analogue scale (VAS) of nausea, postoperative length of stay (p-LOS), Identity-Consequence Fatigue Scale (ICFS), and other complications

Detailed information regarding the postoperative ROM is presented in Table 3. Groups B (97.02 ± 2.23, *P* < 0.001) and C (97.36 ± 2.73, *P* < 0.001) showed favourable effects in terms of maximum hip flexion, compared with those of group A (93.80 ± 3.88) on POD3. Such a difference was not observed between groups B and C on POD3 (*P* = 0.58; Table 3).

The ICFS scores on POD3 were significantly lower for group C (66.58 ± 8.89) than for group A (76.46 ± 10.13, *P* < 0.001) and B (71.06 ± 9.19, *P* = 0.02). This difference was also observed between groups A and B on POD3 (*P* = 0.005; Table 3).

The p-LOS of groups A, B, and C were 5.10 ± 0.54, 4.98 ± 0.43, and 4.92 ± 0.60 d, respectively. There were no significant differences among them (*P* = 0.23; Table 3).

As shown in Table 3, there were two patients with poor wound healing in groups A and C and three patients in group B. There were no significant differences among the groups (*P* = 0.86). No SSI or GIB was observed in our study.

### Degree of satisfaction

As shown in Table 4, four grades of satisfaction were used: Wonderful (A), Good (B), Not Bad (C), and Terrible (D). Patients with grades A and B rated their treatment satisfactorily. The degrees of satisfaction in groups A, B, and C were 76%, 86%, and 94%, respectively. No significant differences were observed between the three groups. However, the degree of satisfaction in group C was significantly higher than that in group A. However, this difference was not detected between groups A and B, or between groups B and C.

**Table 4:** Satisfaction degree.

	A	B	C	D
Group A	30	8	7	5
Group B	35	8	4	3
Group C	40	7	3	0
χ^2^/*P*	8.12/0.23			
χ^2^/*P*1	1.70/0.64			
χ^2^/*P*2	8.10/0.04			
χ^2^/*P*3	3.54/0.32			

*P*: A versus B versus C; *P*1: A versus B; *P*2: A versus C; *P*3: B versus C.

A: Wonderful; B: Good; C: Not bad; D: Terrible.

## Discussion

DEX, a long-acting glucocorticoid with strong anti-inflammatory characteristics, is widely used to reduce perioperative inflammatory response, relieve postoperative pain and fatigue and reduce the incidence of PONV^13,14^. Some randomised controlled trials and meta-analyses have demonstrated the efficacy of DEX in preventing inflammatory stress, with no reports of increased complications, such as SSI or GIB after THA^5,14–16^. However, postoperative pain sometimes occurs even after the administration of a dose of DEX after 24 h^9^. Based on these findings, we considered that an additional dose of DEX may still be required after 48 h.

Postoperative hypothermia is a common complication that is easily ignored after surgery. This hypothermia is often caused by operating room hypothermia, anaesthesia, infusion, or surgery^17,18^. In postoperative hypothermia, core body temperature after anaesthesia generally ranges from 32 to 36 °C, but its incidence has been rarely reported and fluctuates markedly from 20 to 74%^19,20^. Hypothermia disrupts and aggravates the coagulation mechanism, increases perioperative blood loss and the risk of blood transfusion, reduces the postoperative recovery rate and degree of patient satisfaction, and even increases the risk for postoperative death^17,21,22^. The effect of DEX combined with aggressive warming has not been mentioned in the accelerated rehabilitation surgery strategies for THA. We hypothesised that a repeat dose of DEX at 48 h postoperatively along with aggressive warming during surgery may reduce postoperative fatigue, accelerate postoperative recovery, and improve patient satisfaction.

Common complications of THA include pain and PONV. Moderate-to-severe postoperative pain, which is most prevalent during the first three days after surgery, is closely related to inflammatory responses^23^. DEX can effectively reduce the postoperative inflammatory response and postoperative pain^9,24,25^. However, only the first 24 h after surgery have been examined in most of the studies^9,24^. Lei et al.^9^ conducted a prospective randomised controlled trial in patients who received THA. DEX (10 mg) administered at the beginning of anaesthesia and 24 h after anaesthesia effectively reduced postoperative inflammatory response and pain. However, many patients still had obvious pain and other discomfort on the third day after surgery. In a prospective study conducted in The West China Hospital of Sichuan University, repeated doses of DEX up to 48 h could further reduce pain and inflammation after THA; further, repeated application of DEX (10 mg) at 48 h after THA on POD3 was found effective^14^. In the present study, postoperative inflammation and pain levels of patients in group B were significantly lower than those in group A, thus confirming the effectiveness of repeated administration of DEX (10 mg) 48 h following surgery. However, we failed to detect a difference in the pain levels between group A and B participants at rest on POD3. We believe this could be attributed to the generally lower pain score at rest on POD3; the pain perception of the patients might not have been sensitive enough to detect the potential relief of pain at rest. PONV is a common complication after THA that affects the degree of patient satisfaction, delays postoperative recovery, and increases psychological and economic burdens^26^. In this study, no difference in PONV was detected among the three groups after surgery. The most probable reason for this would be that most cases of PONV occurred within 24 h after surgery, consistent with the observations in a previous study^27^. Therefore, the repeated application of DEX at 48 h may have had a limited effect on PONV reduction.

Thermal intervention has been indicated to effectively reduce perioperative blood loss, thus reducing the blood transfusion rate^17^. In a prospective trial, Reina et al.^28^ observed over 900 patients who underwent THA to explore the effects of tranexamic acid (TXA) under mild hypothermia and recorded the occurrence and frequency of blood transfusions and associated complications. The incidence of hypothermia in THA was as high as 84.2%; however, mild hypothermia did not affect the efficacy of TXA, which effectively reduced perioperative blood loss and blood transfusion rate. Winkler et al.^29^ studied the changes in core temperature, blood transfusion rate, and length of hospital stay in 143,157 patients during THA. The core temperature decreased during the first hour and increased thereafter. Nearly 50% of the patients had a low core temperature below 36 °C, while that of 20% of the patients was below 35.5 °C for more than 1 h. Moreover, 20% of patients had a core temperature below 36 °C, among which 8% had a core temperature below, 35.5 °C for more than 2 h. Kurz et al.^19^ reported that maintaining normothermia intraoperatively probably decreased the incidence of infectious complications in patients undergoing colorectal resection and shortened their hospital stay. Hypothermia is a normal phenomenon during the first hour after anaesthesia even at high body temperatures, which confirms that hypothermia would increase the transfusion rate, and aggressive warming could reduce blood loss during THA and reduce complications. In our study, the average temperature of patients in group C was significantly higher than that in groups A and B at 30 and 60 min after the operation and at the end of the operation, because of thermal intervention, thus confirming the effectiveness of the thermal insulation measures. We statistically analysed the IBL, PDV, and transfusion rates of the patients in the three groups. The IBL and PDV of patients in group C were (252.00 ± 43.43) mL and (421.30 ± 44.34) mL, respectively, which were significantly lower than the corresponding values of patients in groups A and B (*P* < 0.001), consistent with the findings from previous studies^17,29^. Therefore, we conclude that warming intervention is effective in reducing IBL and PDV in THA. However, no difference was found in blood transfusion rates among the three groups. This could be attributed to surgical improvements, choice of anaesthesia technology, and optimal management of blood conservation during the perioperative period of THA. Owing to these aspects, the blood transfusion rate of conventional THA has gradually decreased, and no difference could be detected among the three groups.

ROM, p-LOS, and ICFS can be important reference indicators to comprehensively reflect the postoperative recovery rate of patients^13^. Improving patient satisfaction is an urgent issue to resolve. In a meta-analysis of the relationship among pain, sleep, and fatigue, Whibley et al. ^30^ pointed out that these three factors influence and interact with each other. Only when each factor is positively controlled can a virtuous circle be established to promote early recovery of patients, shorten the average length of stay, and improve their degree of satisfaction. In our study, ROM and ICFS of patients in group B were superior to the values of patient in group A on POD3 (*P* < 0.05). Thus, we could administer an additional dose of DEX at 48 h to effectively relieve fatigue and improve the ROM. By comparing groups B and C, we conclude that DEX combined with aggressive warming can effectively relieve postoperative fatigue and improve short-term ROM. Based on this conclusion, we considered that the use of aggressive warming during the operation reduced the incidence of postoperative hypothermia and chills as well as perioperative blood loss, thus improving the recovery speed of the body and the initiation of postoperative rehabilitation of patients^29^. However, there was no significant difference in the p-LOS among the three groups; nevertheless, we presume that the stay was short enough that the effect of an additional dose of DEX was not obvious. In addition, the satisfaction degree of patients in the three groups did not differ significantly (χ^2^ = 8.12, *P* = 0.23); thus, one additional dose of DEX may not significantly improve the satisfaction of patients (group A vs. B). However, based on aggressive warming, adding an additional dose of DEX at 48 h did significantly improve patient satisfaction unexpectedly (group A vs. C, χ^2^ = 8.10, *P* = 0.04). Therefore, aggressive warming can be deemed a necessary procedure.

In our study, no serious complications, including SSI or GIB, occurred in any patient. However, the potential for SSI and GIB with DEX use is not negligible^31^. Hannon et al.^32^ performed a meta-analysis that demonstrated that single or multiple doses of intravenous DEX help reduce postoperative pain, opioid consumption, and PONV. However, the analysis highlighted the insufficient evidence on the risk of postoperative adverse events. Owing to the relatively small sample size (50 cases in each group) and short follow-up time (3 months), our study may lack sufficient strength to measure events that are infrequent^33^. Therefore, our findings should be interpreted with caution, and further large-scale prospective studies are necessary.

This study has some limitations. First, the follow-up time was too short to adequately assess the efficacy and safety of DEX after the three-month-follow-up. Second, as mentioned, we included 50 patients in each group and the small sample size weakened the persuasive power of the study. Third, the complications in this study focused only on SSI and GIB. Other complications, such as blood glucose changes, were not closely followed. Finally, there is a scope for errors during axillary temperature measurements.

## Conclusion

In summary, we conclude that one additional dose of DEX at 48 h has short-term advantages in terms of improved analgesic and anti-inflammatory effects with accelerated rehabilitation after THA without increased chance for complications. DEX combined with aggressive warming can further optimise short-term ROM and fatigue and thus improve the degree of patient satisfaction.

## Data Availability

The datasets used and analysed during the current study available from the corresponding author on reasonable request.
